# Death of Dr. Pean, of Paris

**Published:** 1898-02-05

**Authors:** 


### DEATH OF DR. PEAN, OF PARIS.

We regret to have to record the death of Dr. Pean, of Paris, which resulted from a sudden attack of infectious pneumonia. Dr. Pean was a man of great originality, full of ideas, a powerful teacher, a good surgeon, and a bold and skilful operator. He was a general surgeon in the full sense of the word, but of late years his name has been largely associated with abdominal and pelvic operations. He was one of the first, perhaps actually the first, to do ovariotomy in France, his name is widely known in connection with the use of pressure forceps for the arrest of hæmorrhage, and he was the introducer of the method of removing the uterus by way of the vagina for the cure of chronic inflammation of the appendages, an operation which has in other hands developed into panhysterectomy with the same object.

